# Supplementary material for: Biomaterial-associated molecular patterns (BAMPs) modulate macrophage polarization in bone grafting
Source: PLoS One. 2026 Apr 21;21(4):e0345787. doi: 10.1371/journal.pone.0345787 (PMC13098981; doi:10.1371/journal.pone.0345787)
Supplement: S1 Table — (DOCX) [file pone.0345787.s004.docx]

| **Fluorochrome** | **Antibody (Surface Marker)** | **Manufacturer** |
| --- | --- | --- |
| **APC/Cyanine7** | CD11b | BioLegend  Cat: 101225 |
| **PE-CF 594** | CD14 | BD Biosciences  Cat: 562335 |
| **Alexa 700** | CD16 | BD Biosciences  Cat: 557920 |
| **PE** | CD86 | BD Biosciences  Cat: 557344 |
| **APC** | CD206 | BD Pharmingen  Cat: 561763 |
| **PE-Cy7** | HLA-DR | BD Biosciences  Cat: 560651 |
