# Supplementary material for: Biomaterial-associated molecular patterns (BAMPs) modulate macrophage polarization in bone grafting
Source: PLoS One. 2026 Apr 21;21(4):e0345787. doi: 10.1371/journal.pone.0345787 (PMC13098981; doi:10.1371/journal.pone.0345787)
Supplement: S2 Table — (DOCX) [file pone.0345787.s005.docx]

| **Cytokines** | **ELISA kit** |
| --- | --- |
| **Human IL-1β (Interleukin 1 Beta)** | Elabscience- E-EL-H0149_48tests |
| **Human IL-10 (Interleukin 10)** | Elabscience- E-EL-H6154_48tests |
| **Human TNF-α (Tumor Necrosis Factor Alpha)** | Elabscience- E-EL-H0109_48tests |
| **Human TGF-β1 (Transforming Growth Factor Beta 1)** | Elabscience-E-EL-H0110_48tests |
