# Supplementary material for: Biomaterial-associated molecular patterns (BAMPs) modulate macrophage polarization in bone grafting
Source: PLoS One. 2026 Apr 21;21(4):e0345787. doi: 10.1371/journal.pone.0345787 (PMC13098981; doi:10.1371/journal.pone.0345787)
Supplement: Attachment — Submitted filename: Response to Reviewers.docx [file pone.0345787.s006.docx]

**Date: 26.11.2025**

**Pradeep Kumar, Ph.D.**

Academic Editor
PLOS ONE

Dear Dr. Pradeep,

We sincerely thank you and the reviewers for the constructive and valuable comments on our manuscript titled **“*Distinct biomaterial-associated molecular patterns (BAMPs) associate with distinct macrophage polarization and immune responsiveness in the context of bone grafting*” with *Manuscript ID:*** *PONE-D-25-46093*. We greatly appreciate the time and effort dedicated to reviewing our work.

In accordance with the reviewers’ suggestions, we have thoroughly revised the manuscript. As requested by **Reviewer #3, we have updated the manuscript title to “Biomaterial-Associated Molecular Patterns (BAMPs) Modulate Macrophage Polarization in Bone Grafting” to reflect the study's scope and findings better**. Please find below a detailed, point-by-point response to all reviewer comments. We believe the revisions have strengthened the quality, clarity, and scientific rigor of the manuscript.

| Comments | Response |
| --- | --- |
| Academic Editor comments |  |
| 1. Please ensure that your manuscript meets PLOS ONE's style requirements, including those for file naming. | Thank you for your comment. The manuscript has been prepared in accordance with the PLOS ONE style templates. |
| 2. Please include a separate caption for each figure in your manuscript. | We appreciate your attention and thank you for informing us. A caption has been provided for each figure in accordance with the PLOS ONE style template. |
| 3. If the reviewer comments include a recommendation to cite specific previously published works, please review and evaluate these publications to determine whether they are relevant and should be cited. There is no requirement to cite these works unless the editor has indicated otherwise. | We once again thank the academic editor for the important comment. As noted by Reviewer 4, the referenced publication has already been cited in our initial manuscript submission. Our previously published work complements the current study; therefore, we have incorporated several sentences discussing its relevance in the introduction and discussion sections, underscoring its connection to the present work. |
|  |  |
| Reviewer #1:This study investigated the physicochemical properties and adsorbed serum proteomic profiles of demineralized (DMB) and decellularized (DCC) bone grafts, as well as the immunomodulatory effects of serum proteins adsorbed onto these grafts on the phenotypic and functional profiles of macrophages. The work is well-conceived and supported by relevant literature. However, several issues need to be addressed before the manuscript can be considered for publication. | Thank you for your time and effort in reviewing our study. We appreciate your comments on the data presentation, and we have revised it accordingly. We also thank you for your remarks on our research, which you found well-conceived and supported by relevant literature. |
| 1. First, the manuscript contains grammatical errors and requires thorough revision to ensure consistency in the use of standard scientific English (e.g., coverslip vs. cover slips). | We appreciate the reviewer for highlighting this point. The manuscript has been thoroughly revised and proofread multiple times. Grammarly (v1.2.177.1709) was used to enhance readability and language, ensuring adherence to U.S. English standards. |
| 2. Second, the description of critical methodological steps, particularly the protein adsorption and desorption processes from bone grafts and the THP-1 cell culture conditions, is insufficient and needs to be expanded for reproducibility. | We understand the importance of this comment. Therefore, we have included detailed methodological steps for protein adsorption and desorption, as well as for cell treatment. Additionally, we have included detailed supplementary figures illustrating the protein adsorption and desorption process and cell treatment methodology in the manuscript.  Please find the details in Materials and Methods  Section: 2.3.1, Pg: 6-8, Line: 162-194.  Section: 2.4.3 Pg: 10, Line: 240-254.  Figures: S1 Fig, S2 Fig |
| 3. In addition, the authors are encouraged to include correlation analyses for the evaluated markers to strengthen the interpretation of their results. For instance, assessing associations between LPS stimulation and iNOS production in DMB samples, or between IL-4 treatment and Arg-1 expression in DCC samples, would provide valuable mechanistic insight. | We appreciate the reviewer's suggestion to include additional statistical analyses. However, in this study, the methods used and data collected were based on a single concentration and time point. As a result, the study design does not support robust correlation analysis, which generally requires experimental conditions with different concentrations or time points to evaluate associations accurately. Consequently, correlation analysis was not conducted in this work. We plan to include experimental designs with varying concentrations in future studies to facilitate comprehensive correlation analysis. |
| 4. The presentation of cytokine quantification should also be revised: the units (e.g., pg/mL) must be reported consistently with the values shown in Figure 6. | Figure 6 has been renumbered as Figure 7.  We would like to draw your kind attention to the details in Figure 7. The upper set of histograms, from (A) to (D), shows mRNA expression of cytokines relative to GAPDH. Meanwhile, the lower sets of histograms, from (E) to (H), show the protein expression in pg/ml. We made a slight modification to this figure by adding a line between the two sets and including a small heading to improve visibility, indicating that the upper histograms represent mRNA expression and the lower histograms show protein concentration (pg/mL).  Please find the revised Figure 7. |
| 5. Although IL-10 levels were lower compared to other cytokines, this molecule is a key immunoregulatory factor. The authors are strongly advised to explore the biological processes related to IL-10 and discuss its relevance in the context of their findings. | We appreciate the reviewer's insightful comment. Indeed, IL-10 is a critical immunoregulatory cytokine, and its role in bone graft integration, as described by our data, deserves further discussion. That said, and not to overstate the significance of IL-10 in bone grafting and regeneration, we included a brief paragraph further discussing the broader relevance of IL-10 in relation to our findings.  Please see the discussion section.  Section: 4, Pg: 25, Lines 627-645 |
|  |  |
| Reviewer #2: The manuscript describes an interesting and novel study, but it cannot be accepted in its current version. A minor revision is recommended, considering the following comments: | We appreciate the reviewer's comments and are pleased that the reviewer found the study both innovative and interesting. We also thank you for your suggestion of minor revisions. |
| 1. The introduction must include the differences between decellularized and demineralized tissues, as well as a justification for their use in this study. | Thank you for pointing this out to us. We have added a brief paragraph outlining the differences between decellularized and demineralized tissues, along with a justification for their use in this study.  Please see the introduction section,  Section: 1, Pg: 4, Lines: 95-106 |
| 2. The approval of the ethics committee for conducting this study must be included in the manuscript | We used bovine bone samples obtained from a certified slaughterhouse, and the THP-1 cell line was acquired as described in the methodology. Based on our materials and methods, ethical approval from the ethics committee is not required. We also acknowledge the importance of this comment. Therefore, we are providing a letter stating that this study does not necessitate ethical approval from the University of Sharjah Animal Care and Use Committee.  Please find the attached file titled Ethical Committee Letter. |
| 3. In Section 2.2.3, why was SEM chosen for sample analysis if magnifications of 60x and 100x can be achieved with an optical microscope? | We appreciate the reviewer's comment on the details of the results. While magnifications of 60× and 100× are achievable with standard optical microscopy, we chose Scanning Electron Microscopy (SEM) for its superior surface clarity and structural resolution. Optical microscopy was initially insufficient for clearly delineating the scaffold architecture needed for accurate roughness measurement; in contrast, SEM provided enhanced depth of field, contrast, and surface detail in high-quality micrographs. As a result, SEM images at 60× and 100× magnifications were deemed more reliable for assessing surface roughness using ImageJ. We previously mentioned in the initial manuscript submission that SEM images were used for surface roughness analysis in ImageJ software.  Please see the materials and methods section,  Section: 2.2.3, Pg: 6, Lines: 158-159 |
| 4. How were the time intervals chosen in Section 2.3.1? | The time interval was chosen based on a previously published article about time-dependent protein adsorption on surfaces. However, the initial and final time points corresponded to the early phase of protein adsorption relevant to the FBR process in bone grafts. The time interval reference is provided in the methodology section.  Please see the Materials and Methods section.  Section: 2.3.1, Pg: 7, Lines: 166-173 |
| 5. In Section 2.4.2, are the DMSP and DCSP concentrations proposed by the authors or based on previous studies? | Thank you for highlighting this point. We have included the reference from the previous study that explains the treatment concentration of the extracellular matrix protein.  Please see the methodology section, Section: 2.4.2, Pg:9, Lines: 233-234 |
| 6. The method section must include references to the concentration and conditions evaluated if they are based on previous studies. | Thank you for informing us. We have established the concentrations and conditions based on our experimental results. The concentrations were chosen based on our XTT assay results.  Section: 3.6, Pg: 18, Lines 441-444 |
| 7. The references should be updated to the latest ones | Thank you for bringing it to our attention. We have revised the reference accordingly.  Please find the reference section. Pg 27-34 |
|  |  |
| Reviewer #3: The manuscript is well-written, with clear structure and logical flow. The abstract effectively summarizes the study, and the discussion contextualizes findings within existing literature. However, the authors should consider revising the following: | We appreciate the reviewer's efforts and the time dedicated to evaluating the manuscript. We also acknowledge your comments that the manuscript is clear, well-structured, and logical in its presentation. |
| 1. The title is quite long and somewhat repetitive (e.g., "distinct" appears twice). The authors may consider this; "Biomaterial-Associated Molecular Patterns Modulate Macrophage Polarization in Bone Grafting."   Ensure consistent formatting of figures and tables (some legends are embedded in the text). | Thank you for suggesting the new title. We have revised it accordingly and are pleased to implement the proposed change. Additionally, we have informed the academic editor about this update.  Thank you for informing us. We have amended and reformatted the figures and tables in accordance with the PLOS ONE style template. |
| 2. Show Scale bars in the images, what is there are not legible. | Thank you for pointing this out to us. We have improved the clarity of the figure, so the scales are more visible.  Please see the figures attached. |
| 3. Clarify acronyms on first use (e.g., DMSP, DCSP). | Thank you. We have corrected it accordingly.  Please see the materials and methods section,  Section: 2.3.1, Pg: 8, Line 191 |
|  |  |
| Reviewer #4: Brigi et al present a study of absorbed proteins on bovine based bone grafts incubated with THP-1 cells and review the effects of the bone surface morphology and chemistry on CD14, CD16, CD86, CD206, IL-1beta, TBF-alpha, etc. Two forms of the bone substrate were investigated: demineralized bone graft (BMD) and decellularized bone graft (DCC). The authors strive to investigate the effect of the graft chemistry and morphology on the profile of absorbed proteins from FBS and their subsequent immunomodulatory effect on macrophages. Overall I find the presented work thorough and well devised. The statistical analysis is adequate. Based upon the submitted manuscript and the comments below, I recommend revision prior to publication. | We thank the reviewer for the comments that could improve the detail of this manuscript. We also appreciate the positive comments recognizing the work as thorough and well devised, and for recommending revision before publication. |
| 1. In section 2.1, the authors mention that the substrates undergo a sterilization process. No details of this process are given. Please include further details. | Thank you for bringing that to our attention. We have included the details of the sterilization process in section 2.1.  Please see the materials and methods section.  Section: 2.1, Pg: 5, Lines 124-125 |
| 2. In section 2.2.1, how were the samples prepared for water contact angle measurements? Were bone sections prepared? Or were the original surfaces used? | Bone samples were not sectioned, and the original surface was used for the contact angle measurements. We have included a statement regarding this.  Please see the materials and methods section.  Section: 2.2.1, Pg: 5, Lines 130-131 |
| 3. In section 2.2.2, the bone samples were prepared into a fine powder. How was the bone powder prepared, and to what nominal particle size for the zeta potential measurements? | Thank you for bringing this to our attention. These details are included in the methodology section, where the bone sample preparation, method, and instrument used are specified. Additionally, the nominal particle size for zeta potential is provided in the results section.  Please see the materials and methods section.  Section: 2.2.2, Pg: 5,6, Lines 138-145  Please see the result section.  Section: 3.2, Pg: 14, Lines 338-339 |
| 4. Based upon the work of the authors cited in Ref 30, I find this work to be a complement to the 2024 study. Unfortunately the work from e0300331 are not included in the discussion or introduction. The previous work focused on macrophage polarization but the present work strives to elucidate the substrate surface chemistry and protein absorption profile's effect on macrophage differentiation. | We thank the reviewer for this insightful and in-depth consideration of the exact scope of our study. We briefly describe, in the discussion and introduction, how the present study complements our previous work.  Please see the introduction section.  Section: 1, Pg: 4, Lines 107-111  Please see the discussion section.  Section: 4, Pg: 24, Lines 598-607 |

We hope the revisions, including the requested title change, fully address all concerns raised by the reviewers. We are grateful for the opportunity to improve our manuscript and are available for any further clarifications. All authors have approved the revision and agree to submit it to PLOS ONE. We declare no conflicts of interest. Thank you for considering our manuscript for publication. We look forward to contributing to your esteemed journal.

Thank you very much. Warmest regards.

Prof. A.R. Samsudin FDSRCS (England), FICD

Wound Healing & Oral Diagnostics Research Group

Department of Oral and Craniofacial Health Sciences,

College of Dental Medicine,

University of Sharjah, United Arab Emirates

[www.sharjah.ac.ae](http://www.sharjah.ac.ae); [drabrani@sharjah.ac.ae](mailto:drabrani@sharjah.ac.ae); Tel: +971507076556; +97165057600
