# Supplementary material for: Biomaterial-associated molecular patterns (BAMPs) modulate macrophage polarization in bone grafting
Source: PLoS One. 2026 Apr 21;21(4):e0345787. doi: 10.1371/journal.pone.0345787 (PMC13098981; doi:10.1371/journal.pone.0345787)
Supplement: Attachment — Submitted filename: Response_to_Reviewers_auresp_2.docx [file pone.0345787.s007.docx]

**Date: 26.01.2026**

**Pradeep Kumar, Ph.D.**

Academic Editor
PLOS ONE

Dear Dr. Pradeep,

We sincerely thank you and the reviewer for the constructive and valuable comments on our manuscript titled **“Biomaterial-Associated Molecular Patterns (BAMPs) Modulate Macrophage Polarization in Bone Grafting” with *Manuscript ID:*** *PONE-D-25-46093*. We greatly appreciate the time and effort dedicated to reviewing our work.

Please find below a detailed, point-by-point response to reviewer comments. We believe the revisions have strengthened the quality, clarity, and scientific rigor of the manuscript.

| Comments | Response |
| --- | --- |
| Journal Requirements |  |
|  |  |
| 1. If the reviewer comments include a recommendation to cite specific previously published works, please review and evaluate these publications to determine whether they are relevant and should be cited. There is no requirement to cite these works unless the editor has indicated otherwise. | We thank the editorial board for reminding us of this important point. As no specific references were recommended by the reviewer, no changes to the reference list were made in this revision. All references suggested in the earlier revision were thoroughly assessed for relevance, and only those pertinent to the objectives of the present study were incorporated. |
| 2. Please review your reference list to ensure that it is complete and correct. If you have cited papers that have been retracted, please include the rationale for doing so in the manuscript text, or remove these references and replace them with relevant current references. Any changes to the reference list should be mentioned in the rebuttal letter that accompanies your revised manuscript. If you need to cite a retracted article, indicate the article’s retracted status in the References list and also include a citation and full reference for the retraction notice. | The reference list was carefully reviewed for completeness, accuracy, and retraction status. To the best of our knowledge, none of the cited references have been retracted to date; therefore, no changes to the reference list were required. References have been formatted in accordance with the PLOS ONE guidelines. |
|  |  |
| Reviewer #3 |  |
| 1. Ensure consistent tense and terminology (e.g., “cover slip” vs. “coverslip”) | We thank the reviewer for this comment. The manuscript has been revised to ensure consistency in tense and terminology, and the term “coverslip” is now used consistently throughout the text.  Line: 257, 260, 265, 267, 455 |
| 2. Consider shortening the abstract to meet the word limit and improve readability. | We thank the reviewer for this comment. The abstract has been condensed to comply with the PLOS ONE word limit and to enhance readability.  Section: Abstract, Pg: 2, Line: 36-57. |
| 3. Check resolution and legibility of SEM images and scale bars | We thank the reviewer for this comment. SEM images (Fig. 2 & Fig. 6) and figures containing scale bars were enlarged and converted to 600 dpi. All figures were prepared using PLOS’s free figure tool (NAAS) to ensure compliance with PLOS ONE publication-quality figure guidelines. |

We hope the revisions fully address all concerns raised by the reviewer. We are grateful for the opportunity to improve our manuscript and are available for any further clarifications. All authors have approved the revision and agree to submit it to PLOS ONE. We declare no conflicts of interest. Thank you for considering our manuscript for publication. We look forward to contributing to your esteemed journal.

Thank you very much. Warmest regards.

Prof. A.R. Samsudin FDSRCS (England), FICD

Wound Healing & Oral Diagnostics Research Group

Department of Oral and Craniofacial Health Sciences,

College of Dental Medicine,

University of Sharjah, United Arab Emirates

[www.sharjah.ac.ae](http://www.sharjah.ac.ae); [drabrani@sharjah.ac.ae](mailto:drabrani@sharjah.ac.ae); Tel: +971507076556; +97165057600
