# Supplementary material for: Biomaterial-associated molecular patterns (BAMPs) modulate macrophage polarization in bone grafting
Source: PLoS One. 2026 Apr 21;21(4):e0345787. doi: 10.1371/journal.pone.0345787 (PMC13098981; doi:10.1371/journal.pone.0345787)
Supplement: Attachment — Submitted filename: Response_to_Reviewers_auresp_3.docx [file pone.0345787.s008.docx]

**Date: 16.02.2026**

**Pradeep Kumar, Ph.D.**

Academic Editor
PLOS ONE

Dear Dr. Pradeep,

We sincerely thank you and the reviewer for the constructive and valuable comments on our manuscript titled **“Biomaterial-Associated Molecular Patterns (BAMPs) Modulate Macrophage Polarization in Bone Grafting” with *Manuscript ID:*** *PONE-D-25-46093*. We greatly appreciate the time and effort dedicated to reviewing our work.

Please find below a detailed, point-by-point response to reviewer comments. We believe the revisions have strengthened the manuscript's quality, clarity, and scientific rigor.

| Comments | Response |
| --- | --- |
| Journal Requirements |  |
|  |  |
| 1. If the reviewer comments include a recommendation to cite specific previously published works, please review and evaluate these publications to determine whether they are relevant and should be cited. There is no requirement to cite these works unless the editor has indicated otherwise. | We thank the editorial board for reminding us of this important point. As no specific references were recommended by the reviewer, no changes to the reference list were made in this revision. All references suggested in the earlier revision were thoroughly assessed for relevance, and only those pertinent to the objectives of the present study were incorporated. |
| 2. Please review your reference list to ensure that it is complete and correct. If you have cited papers that have been retracted, please include the rationale for doing so in the manuscript text, or remove these references and replace them with relevant current references. Any changes to the reference list should be mentioned in the rebuttal letter that accompanies your revised manuscript. If you need to cite a retracted article, indicate the article’s retracted status in the References list and also include a citation and full reference for the retraction notice. | We thank the editorial board for reminding us of this point. The reference list was carefully reviewed for completeness, accuracy, and retraction status. To the best of our knowledge, none of the cited references have been retracted to date; therefore, no changes to the reference list were required. References have been formatted in accordance with the PLOS ONE guidelines. |
| Reviewer # 3 |  |
| Interpretation of Causality Requires Caution. At several points, the manuscript implies direct mechanistic links between individual proteins and macrophage outcomes (e.g., fibrinogen ↔ M1 polarity; serpin A3 ↔ M2 phenotype). These interpretations are plausible but not demonstrated experimentally. Recommendation: Clarify that these are correlations, not mechanistic conclusions, unless further functional inhibition/knockdown assays are added. | Thank you very much for highlighting our interpretation of the results. We fully agree that our interpretation is merely correlational and not a mechanistic conclusion unless future studies are conducted. Therefore, we have rephrased the text in the respective paragraph to avoid misinterpretation of our claims as follows:  Discussion section; Page: 24,25; Lines: 609-636. |
|  |  |
| Reviewer # 4 |  |
| 1. The authors have attempted to address Reviewer 3's comments regarding the SEM images. However, I find that the scale bars are not legible on the images. It may be necessary to at least state in the legend the value of the scale bar for each image. At 600 dpi the scale bar legends cannot be read. Therefore, I would recommend revision of Figures 2 and 6 and the corresponding captions. | We thank the reviewer for this comment regarding the SEM images (Figures 2 and 6). To preserve image integrity and avoid selective enlargement that could potentially alter visual interpretation, the scale bar values for each panel have now been clearly specified in the revised figure legends. As recommended, the scale bar measurements are explicitly stated for each image, and the figures have been maintained at 600 dpi resolution in accordance with journal guidelines.  Results Section; Page: 18; Line: 457-458  Result Section; Page: 15; Line: 357-360  All figures were prepared using PLOS’s free figure tool (NAAS) to ensure compliance with PLOS ONE publication-quality figure guidelines. |

We hope the revisions fully address all concerns raised by the reviewer. We are grateful for the opportunity to improve our manuscript and are available for any further clarifications. All authors have approved the revision and agree to submit it to PLOS ONE. We declare no conflicts of interest. Thank you for considering our manuscript for publication. We look forward to contributing to your esteemed journal.

Thank you very much. Warmest regards.

Prof. A.R. Samsudin FDSRCS (England), FICD

Wound Healing & Oral Diagnostics Research Group

Department of Oral and Craniofacial Health Sciences,

College of Dental Medicine,

University of Sharjah, United Arab Emirates

[www.sharjah.ac.ae](http://www.sharjah.ac.ae); [drabrani@sharjah.ac.ae](mailto:drabrani@sharjah.ac.ae); Tel: +971507076556; +97165057600
